# Anesthetic protocol for right ventricular dysfunction management in heart transplantation: systematic review, development and validation

**DOI:** 10.1186/s12871-021-01261-5

**Published:** 2021-02-11

**Authors:** Lucas Nepomuceno Barros, Ricardo Barreira Uchoa, Juan Alberto Cosquillo Mejia, Rogean Rodrigues Nunes, Denise Araujo Silva Nepomuceno Barros, Filadelfo Rodrigues Filho

**Affiliations:** 1 State University of Ceará, Fortaleza, Brazil; 2 Dr Carlos Alberto Studart Gomes - Messejana Hospital, Fortaleza, Brazil; 3 Fortaleza General Hospital, Fortaleza, Brazil; 4 Professor in Professional Master’s in Transplants, State University of Ceará, Fortaleza, Brazil

**Keywords:** Heart transplantation, Right ventricular dysfunction, Anesthesia, Protocol

## Abstract

**Background:**

Right Ventricular Dysfunction (RVD) is the most frequent intraoperative hemodynamic complication in Heart Transplantation (HTx). RVD occurs in 0.04–1.0% of cardiac surgeries with cardiotomy and in 20–50% of HTx, with mortality up to 75%. No consensus has been established for how anesthesiologists should manage RVD, with management methods many times remaining unvalidated.

**Methods:**

We conducted a systematic review, following PRISMA guidelines, to create an anesthetic protocol to manage RVD in HTx, using databases that include PubMed and Embase, until September 2018 based on inclusion and exclusion criteria. The articles screening for the systematic review were done two independent reviewers, in case of discrepancy, we consulted a third independent reviewer. Based on the systematic review, the anesthetic protocol was developed. The instrument selected to perform the validation of the protocol was AGREE II, for this purpose expert anesthetists were recruited to do this process. The minimum arbitration score for domains validation cutoff of AGREE II is arbitered to 70%. This study was registered at PROSPERO (115600).

**Results:**

In the systematic review, 152 articles were included. We present the protocol in a flowchart with six steps based on goal-directed therapy, invasive monitoring, and transesophageal echocardiogram. Six experts judged the protocol and validated it.

**Conclusion:**

The protocol has been validated by experts and new studies are needed to assess its applicability and potential benefits on major endpoints.

## Background

Heart transplantations (HTx) present many complications related to the anesthetic and surgical proceedings. Among them, Right Ventricular Dysfunction (RVD) is the most prevalent hemodynamic complication in the intraoperative and postoperative periods [1]. RVD occurs in up to 20–50% of cases [2]. RVD is one of the most severe complications to occur during the intraoperative period [3]. It’s a frequent complication following general heart surgeries, much more difficult to treat than left ventricular dysfunction [4]. Acute RVD after cardiac surgery is associated with mortality rates as high as 75% [5].

Many risk factors contribute to the development of acute RVD, such as acquired or preexistent Pulmonary Hypertension (PH), multiple redo operation, re-cross clamping and arresting the transplanted heart, suboptimal intraoperative myocardial protection (stunning), coronary embolism or graft occlusion causing RV (Right Ventricle) ischemia [5, 6], mechanical obstruction at the anastomosis of the pulmonary arteries, significant size mismatch (> 20%), acute graft rejection, ischemic time, adverse reactions and hypersensitivity to drugs [7].

Patients with risk factors or previously known RVD have hemodynamic monitorization commonly reported via Pulmonary Arterial Catheter (PAC) in up to 87% and Transesophageal Echocardiogram (TEE) up to 74% of cases. Perioperative monitoring with a PAC (Swan-Ganz catheter) presents hemodynamic parameters that aid in the diagnosis of RVD, such as a pulmonary vascular resistance [8]. Additionally, one of the most common tools used to evaluate the RV is the TEE, where variables such as chamber volumes, Fractional Area Change (FAC), and Ejection Fraction of the RV (RVEF) can be assessed [9–11].

This study proposes to develop and to validate a proper protocol for anesthetic RVD management in HTx based on recent publications to standardize anesthetic conduct in the face of impending RVD leading to significant hemodynamic consequence during HTx.

## Methods

This study consists of three phases: systematic review, development and validation of the protocol. This study was approved by Research Ethics Committee of the Hospital de Messejana Dr. Carlos Alberto Studart Gomes (CEP-HM), accredited by the National Research Ethics Commission of the National Health Council of the Ministry of Health (03993218.5.0000.5039). All methods were performed in accordance with the relevant guidelines and regulations, including, but not limited to, PRISMA, PROSPERO, PICO, AGREE II.

### Systematic review

We conducted a systematic review of major points regarding RVD management in HTx for the protocol. We used databases from Scielo, Lilacs, PubMed, Capes/MEC, Embase and Clinicalkey, and the study was registered at PROSPERO (115600). We framed the PICO (Population/Patient/Problem, Intervention, Comparison, Outcome) question [12]: “How should the anesthesiologist manage the RVD in HTx?” as research question. Search formulas were composed by MeSH terms “heart transplantation”, “right ventricular dysfunction”, and “pulmonary hypertension”. We screened all citations through September 2018 published in English, French, Portuguese and Spanish (inclusion criteria). Publications involving pediatric patients or animals were excluded (exclusion criteria). PRISMA guidelines were followed [13].

Two independent reviewers (LNB and DASNB) screened titles and abstracts of all citations from the initial search result. Then, we followed with a full-text review of the articles that met inclusion criteria on preliminary screening to determine the eligibility of the articles for data extraction. Then exclusion criteria were applied. References of preliminary articles were read in full to recruit new relevant publications. In case of any discrepancy, a third reviewer (BAS) was consulted.

### Protocol development

The protocol was developed based on recent evidence found in literature and seeks to elucidate major points of heterogeneity and discrepancy in anesthesiologist conduct. It’s proposed an anesthetic protocol in a six steps flowchart based on goal-directed therapy, invasive monitoring, and TEE. Five regular anesthesiologists (general target audience) with HTx experience in a large transplantation center were consulted – from December 2018 to January 2019 – regarding their opinions, criticisms, and suggestions about the protocol.

### Protocol validation

After ethics committee approval the protocol was submitted to appreciation of expert anesthesiologists (judges). It was chose the AGREE II^14^ as the validation process instrument, which is generic and can be applied to protocols, guidelines, and any step of human care, including aspects related to public health, screening, diagnosis, treatment or interventions. AGREE II has been translated into many languages, has been cited in over 600 publications, and is endorsed by several health care organizations [14, 15].

AGREE II consists of 23 key items organized within six domains followed by two global rating items (“overall assessment”). Each domain captures a unique dimension of guideline quality: Scope and Purpose, Stakeholder Involvement, Rigor of Development, Clarity of Presentation, Applicability, and Editorial Independence. Our overall assessment includes the guideline quality rating and whether the guideline should be recommended for use in practice. Each of the AGREE II items and the two global rating items are rated on a 7-point scale (1–strongly disagree to 7–strongly agree). AGREE II recommends that each guideline is assessed by at least 2 appraisers, preferably 4, as this increases the reliability of the assessment.

It is of great importance that the validation judges are experts in the field, aiming at an adequate and reliable evaluation of the process. First, it is necessary to consider that there is no consensus on a minimum profile on how to characterize an expert. However, scoring systems, such as that of Joventino’s [16], have been created and establish a minimum score of 5 points as a cut-off point from the sum of the following criteria: PhD degree, 4 points; specific area PhD dissertation, 2 points; master’s degree, 3 points; specific area thesis, 2 points; specific area indexed journal article published, 1 point; recent professional experience (clinical, teaching, research) of at least 5 years on the specific area, 2 points; specific area specialist degree, 2 points. The specific area chosen to be the scope of this study is heart transplantation, anesthesia for heart transplantation or right ventricular dysfunction.

This study aims to be developed by and focused on the anesthesiologist. Therefore, anesthesiologists with experience on HTx that potentially meet the expert criteria were contacted by email, telephone and social media. In total, 17 invitation letters were sent to these potential experts. The sampling was defined as the number of experts that successfully responded to the invitation in a period of 6 months as long as they meet the minimum AGREE II guidelines [14].

For the validation process, AGREE II uses a form that sums up all the scores (which are grouped into domains) for the individual items and scales the final result as a percentage of the maximum possible score for that domain [14]. Among multiple possible scoring interpretations, we arbitrate as high-quality domains those with scores > 70%. Based on the domain scores and experts’ suggestions, we have improved the protocol to meet the needs and expectations of anesthesiologists.

## Results

### Systematic review

**Fig. 1:**
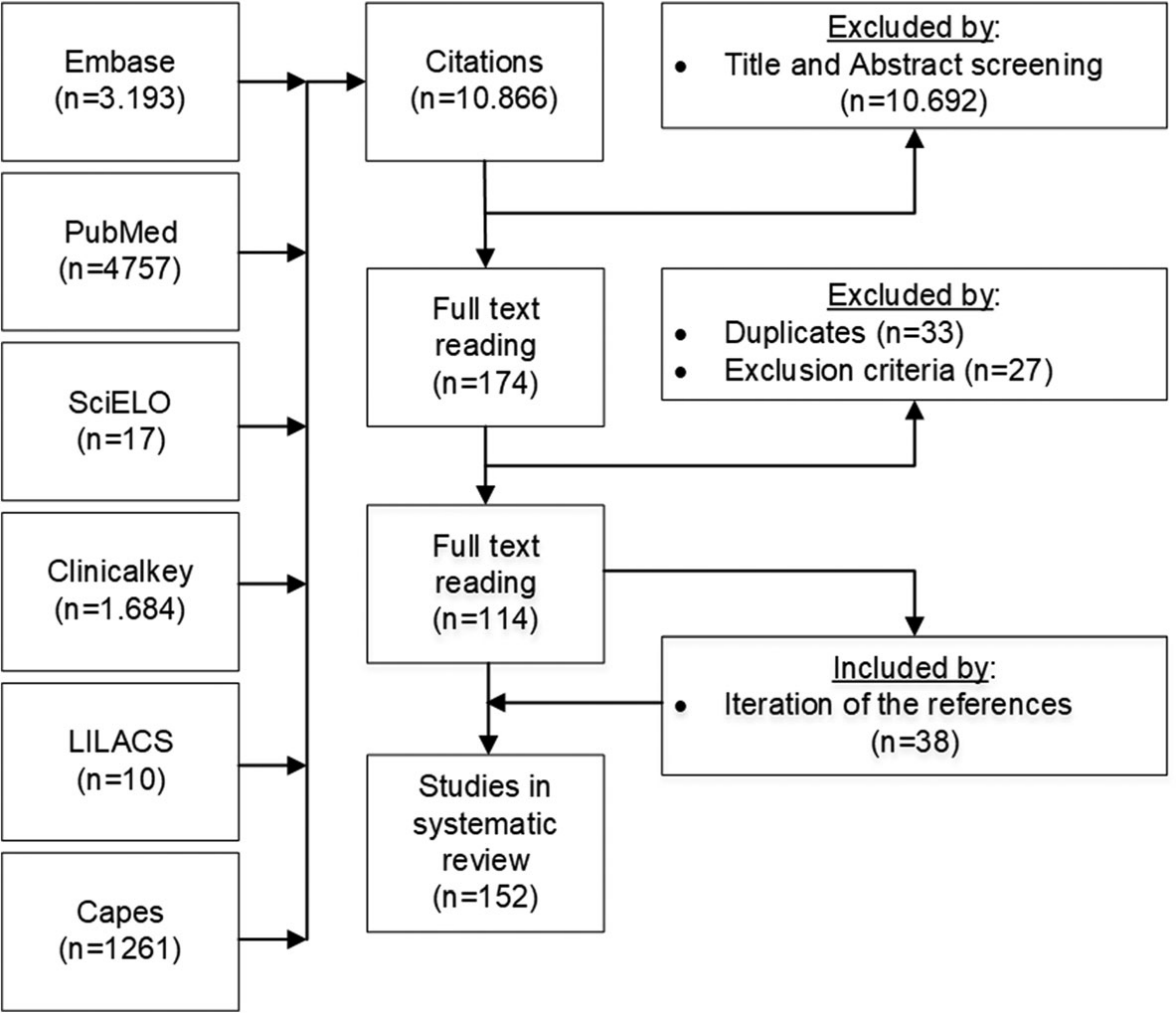
Prisma flowchart demonstrating study selection process

Overall, 10.866 citations met the search equation, of which 10.692 underwent elimination by title and abstract screening. Full text screening was performed on 174 articles, of which 27 met exclusion criteria and 33 were duplicates. An additional 38 articles were summed by iteration of references, providing a grand total of 152 articles for inclusion (Fig. 1).

HTx is a therapeutic option for patients with end-stage heart failure [17]. The right side of the heart has been historically understudied due to its restricted role in systemic diseases. However, the extraordinary influence of RVD on mortality and morbidity after HTx has increased awareness of the scientific community [18–20]. RVD, PH or both are already present in the recipient in most cases; alternatively, RVD may initiate or be aggravated in various post-implant stages, such as weaning from Cardio Pulmonary Bypass (CPB), protamine administration, hemoderivative transfusion, sternal closure or in the intensive care unit [21–23].

Intraoperative monitoring should be done on multiparameter bases [1] and its described as up to 87% with PAC and up to 74% with TEE [24, 25]. The International Society for Heart and Lung Transplantation (ISHLT) recommends monitoring the following hemodynamic variables in the immediate postoperative period: peripheral oxygen saturation, electrocardiogram, Invasive Arterial Blood Pressure (IBP), Central Venous Pressure (CVP), Pulmonary Arterial Pressure (PAP), Pulmonary Capillary Wedge Pressure (PCWP), cardiac output, and mixed venous oxygen saturation. A bladder catheter should be in place for strict measurement of urine output [26].

TEE is the cornerstone to the intraoperative evaluation of the RV [27] and immediate RVD following HTx can be present in up to 100% of cases, based on Tricuspid Annular Plane Systolic Excursion (TAPSE) [28, 29] parameters.

Attempts to formulate a definition of RVF in terms of absolute hemodynamic values have been confounded by the poor reliability of these measures in defining patients with disproportionate systolic RV function. Further, the echocardiographic assessment of RV size and function is limited. In practice, a combination of clinical and echocardiographic findings is utilized, together with clinical judgment, to recognize this complication.

RVD was defined by the ISHLT as a post-cardiac transplant patient who require RV mechanical support or meet all of the following criteria: CVP greater than 15 mmHg, pulmonary capillary wedge pressure less than 15 mmHg, cardiac index less than 2.0 L/min/m2, and transpulmonary gradient less than 15 mmHg and/or pulmonary artery systolic pressure less than 50 mmHg. In practice, a combination of echocardiographic findings, hemodynamic parameters, direct visual inspection, together with clinical judgment is utilized to recognize this complication.

Typical RVD findings on TEE [5, 30, 31] are: RV base diameter > 41-45 mm, RV medial diameter > 35-40 mm, TAPSE< 17 mm, S′ < 9,5 cm/s, RV FAC < 35% and RVEF< 45%.

Typical RVD findings on PAC [5, 30, 31] are: CVP > 20 mmHg, CVP > PAOP, CI < 2.1 l/min/m^2^.

Typical PH findings on PAC [5, 30, 31] are: RVP > 3woods and PAPm> 35 mmHg.

Typical intraoperative goals [5, 30, 31] are: MAP (Mean Arterial Pressure) ≥60-70 mmHg or 20 mmHg above PAPm, PAPm< 35 mmHg or 25 mmHg below MAP, S_a_O_2_ (Arterial Oxygen Partial Pressure) 96–98%, S_cv_O_2_ (Mixed Venous Oxygen Saturation) > 70%, PVR/SVR (Systemic Vascular Resistance) < 0.66, CI ≥ 2.0–2.2 l/min/m^2^, CVP 8-12 mmHg, PAOP (Pulmonary Artery Occlusion Pressure) 12–15 mmHg, diuresis> 0.5 ml/kg/h, lactate< 3 mmol/l and optimized TEE.

The management of the case should be conducted by an experienced anesthesiologist assigned to the care of the patient, extended pre-oxygenation while avoiding hypoxic pulmonary vasoconstriction reflex associated with hypoxia and hypercarbia. Intravenous inotropes and vasopressors should be started before induction. Judicious fluid administration is required to avoid RV dilation and function worsening [32]. Also, it’s important to avoid nitrous oxide, ketamine, hypoglycemia, hypothermia [26, 33] and air bubbles, which hypertensive pulmonary circulation is especially sensitive to [34], among others.

Mechanical ventilation strategy considers oxygen as a potent pulmonary vasodilator and 100% F_i_O_2_ (Fraction of Inspired Oxygen) should be delivered initially along with gentle mean airway pressures (< 25 cm H_2_O) and low to moderate tidal volumes (< 6 mL/kg) [35]. Expiratory time can be optimized to prevent auto-PEEP (Positive End-Expiratory Pressure) and dynamic hyperinflation while limiting inspiratory pressures [1, 32, 36]. Ongoing careful adjustments of minute ventilation to balance preload [35] and respiratory acidosis should occur in the initial stages of Positive Pressure Ventilation (PPV) [37]. Early drainage of pleural effusions and lung recruitment maneuvers should be considered [1].

RVD cases with hemodynamic stability could be managed with intravenous Phosphodiesterase Type 3 Inhibitors (iPDE-3), Inhaled Prostacyclin (iPC), Nitroglycerin (NTG) and inhaled NO (Nitrous Oxide). RVD leading to significant hemodynamic consequence cases could be managed with norepinephrine (NE) associated with iPDE-3, iPC or dobutamine [24]. Other commonly used drugs include epinephrine, vasopressin and nitroprusside [38].

Mechanical Circulatory Support (MCS) is indicated in decompensated heart failure despite maximal optimization of pharmacotherapy, weaning failure from CPB or acute rejection [39, 40]. Extracorporeal Membrane Oxygenation (ECMO) is the most common modality of MCS used during HTx [41, 42].

### Protocol development

**Fig. 2:**
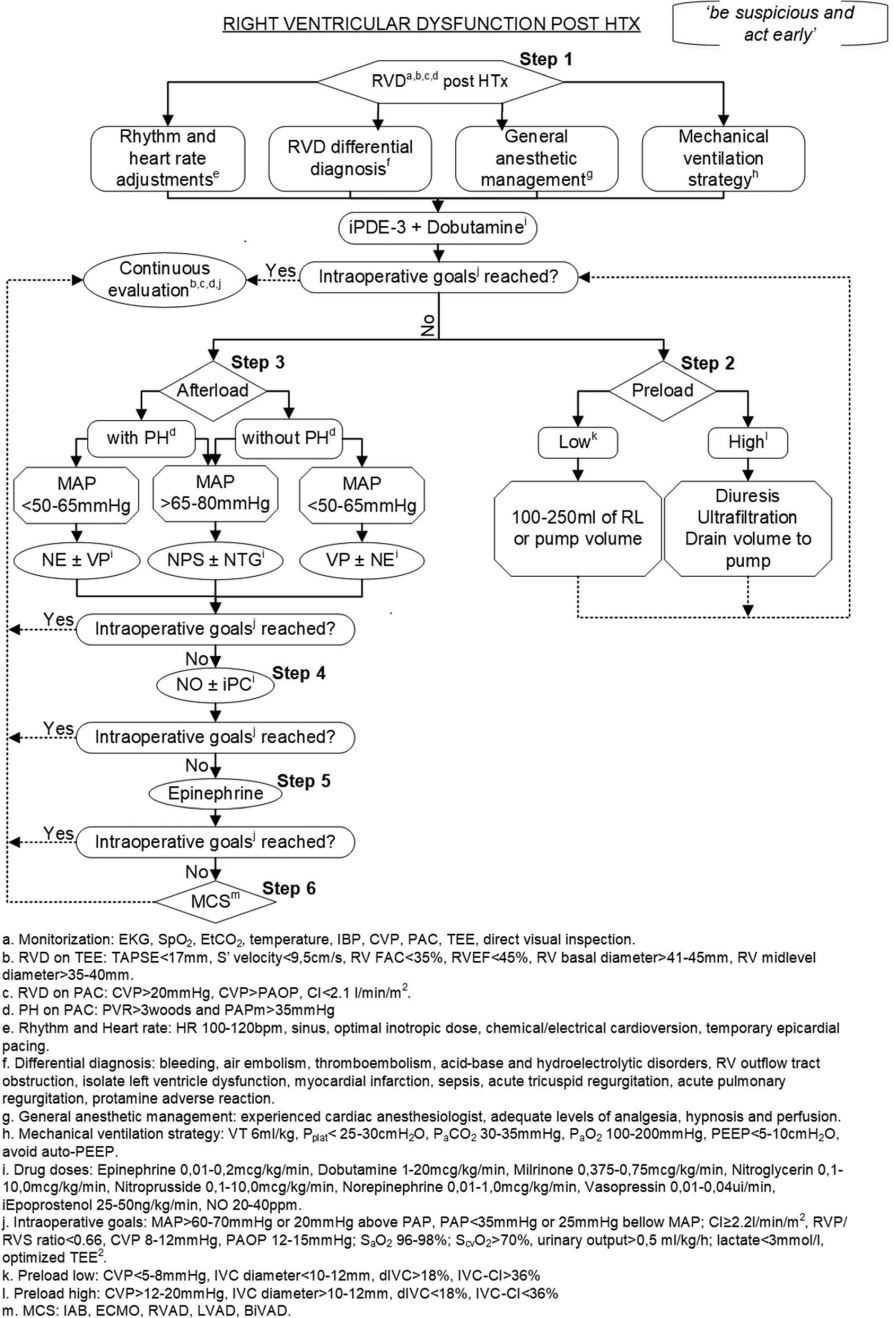
Flowchart for right ventricular dysfunction management in heart transplantation. BiVAD, biventricular assist device; CI, cadiac index; CVP, central venous pressure; CVP, central venous pressure; dIVC, inferior vena cava distensibility index; ECMO, extracorporeal membrane oxygenation; EKG, electrocardiogram; EtCO2, end-tidal carbon dioxide; FAC, fractional area chance; HTx, heart transplantation; IAB, intra-aortic ballon; IBP, invasive blood pressure; iEpoprostenol, Inhaled epoprostenol; iPC, inhaled prostacyclin; iPDE-3, phosphodiesterase type 3 inhibitors; IVC, inferior vena cava; IVS-CI, inferior vena cava collapsibility index; LVAD, left ventricular assist device; MAP, mean arterial pressure; MAP, mean arterial pressure; MCS, mechanical circulatory support; NE, norepinephrine; NO, nitric oxide; NPS, nitroprusside; NTG, nitroglycerin; PAC, pulmonary arterial catheter; P_a_CO_2_, arterial partial pressure of carbon dioxide; P_a_O_2_, arterial partial pressure of oxygen; PAOP, pulmonary artery occlusion pressure; PAP, pulmonary arterial pressure; PEEP, positive end-expiratory pressure; PH, pulmonary hypertension; PH, pulmonary hypertension; P_plat_, plateau pressure; PVR, pulmonary vascular resistance; RL, ringer’s lactate; RV, right ventricle; RVAD, right ventricular assist device; RVD, right ventricular dysfunction; RVEF, right ventricle ejection fraction; S_a_O_2_, arterial oxygen partial pressure; S_cv_O_2_, Mixed venous oxygen saturation; SpO_2_, peripheral oxygen saturation; SVR, systemic vascular resistance; TAPSE, tricuspid annular plane systolic excursion; TEE, transesophageal echocardiography; VP, vasopressin; TV, tidal volume. Source: Elaborated by the author

The philosophy “wait and see” should never be used. Always “be suspicious and act early” [33]. The proposed protocol is presented in the following flowchart (Fig. 2).

This protocol suggests that the Steps should be carried out in a specific order. First, beginning with Step 1, then proceeding simultaneously to both Steps 2 and 3. After this, we can proceed to Step 4, then Step 5, and, finally, to Step 6. If, at any time, the intraoperative goals have been reached, then we proceed to the continuous evaluation stage.

#### Step 1

The protocol begin with exclusion of differential diagnosis that may generate specific treatment conducts, such as surgical bleeding, air embolism, thromboembolism, acid-base and hydroelectrolytic disorders, RV outflow tract obstruction, isolate left ventricle dysfunction, myocardial infarction, sepsis, acute tricuspid regurgitation, acute pulmonary regurgitation [1, 26, 33, 43, 44] while promoting adequate levels of analgesia, hypnosis and perfusion by an experienced anesthesiologist [45], such as a Bispectral Index (BIS) of 40–60 and cerebral rSO_2_ (Regional Cerebral Oxygen Saturation) of 60–75%.

Mechanical ventilation strategy should aim tidal volumes < 6 mL/kg [36], plateau pressure < 25-30cmH_2_O, P_a_CO_2_ 30-35 mmHg, P_a_O_2_ 100-200 mmHg, SpO_2_ 96–98%, PEEP < 5-10cmH_2_O and auto-peep prevention [1, 32, 36].

The implanted graft usually presents some level of RVD demanding a normal-high HR (Heart Rate) and high LV (Left Ventricle) filling pressures to maintain an adequate CO [46]. Concomitant to sinus rhythm, it is desirable to maintain HR of 100–120 [1] through optimization of inotropes, chemical/electrical cardioversion, and/or temporary epicardial pacing.

Intraoperative monitoring should be done on multiparameter bases following ISHLT recommendations with major target goals [5, 31, 47].

The recommended doses of drugs vary greatly in the literature and, through a dynamic interaction with echocardiographic findings, hemodynamic parameters and direct visual inspection, escalation can be done according to the following range: epinephrine 0,01–0,2mcg/kg/min, dobutamine 01-20mcg/kg/min, milrinone 0,375–0,75mcg/kg/min, NTG 0,1–10,0mcg/kg/min, NPS (Nitroprusside) 0,1–10,0mcg/kg/min, NE 0,01–1,0mcg/kg/min, vasopressin 0,01–0,04ui/min, inhaled epoprostenol 25-50 ng/kg/min, and NO 20-40ppm [21, 38].

RVD management is largely empiric and focuses on precipitating factors while optimizing components of RV function such as myocardial contractility, chronotropism, preload and afterload [48]. We should allow reperfusion of the graft for a while, while the myocardial cells restore the ATP cells, and then start inotropic support [22, 24]. After the first inotropic agent start and HR is optimized, we act simultaneously in preload and afterload [9, 48–50].

Once the therapy is optimized, an interrogation of target goals should be done. If intraoperative goals have been reached, we stop advancing on the flowchart and keep constant goal monitoring; otherwise, we proceed to steps 2 and 3 of the flowchart, simultaneously.

#### Step 2

Judicious fluid balance is crucial to successful preload management. If low intravascular volume is suspected by CVP < 5-8 mmHg or Inferior Vena Cava (IVC) diameter < 10-12 mm, IVC distensibility index (dIVC - Inferior Vena Cava Distensibility Index) > 18%, IVC Collapsibility Index (IVC-CI) > 36% on TEE, evaluating the stroke volume response to volume infused from the pump or 100-250 ml warmed ringer lactate solution fluid challenge can be carefully done [51, 52]. A relatively underfilled RV is likely the lesser of two evils [34] and volume overload can lead to catastrophic decompensation on graft RVD.

The most common presentation includes an RV with high preload and can be suspected by CVP > 12-20 mmHg or IVC diameter > 10-12 mm, dIVC < 18%, IVC-CI < 36% on TEE [53, 54]. In this setting, CVP reductions via diuresis, ultrafiltration, or venous drainage into CPB may be followed by an enhanced CO [55].

Fluid challenge should be promptly terminated if the CVP exceeds 12-20 mmHg, CO doesn’t enhance despite preload raisings [48], or PAC shows raisings in PAOP with maintenance or no enhance in CO [34].

After a new conduct has been taken, a new interrogation of intraoperative goals should be made. If intraoperative goals have been reached, we should stop advancing on the protocol flowchart and keep constant goal monitoring; otherwise, we should advance to the next flowchart step.

#### Step 3

Regarding afterload, at this point, inotropic stimulation should be maximally optimized. Therapy with iPDE-3 and dobutamine, which have been started in Step 1, should be associated with norepinephrine and/or vasopressin in hypotensive patients (MAP < 50-65 mmHg). Vasopressin may be considered as first choice for PH patients [22]. In case of a systemic hypertensive patient (MAP > 65-80 mmHg) we may proceed with vasodilation by using NPS and/or NTG [38].

#### Step 4

Inhaled pulmonary vasodilators (e.g., NO, prostacyclin) should be associated in case of worsening hemodynamic parameters despite optimal intravenous therapy, previous PH and/or RVD refractory to intravenous drugs [21]. Special attention should be given to the intraoperative goal and it should not limit itself to a normal PVR or PAOP, but instead to an optimization of PVR/SVR ratio, maintaining myocardial contractility and maximizing DO_2_ (Delivery of Oxygen).

#### Step 5

In case of maximal vasoactive and the inotropic therapy associated with pulmonary vasodilators fails, a new potent inotropic (epinephrine) can be instituted in an attempt to enhance CO [31].

#### Step 6

If ventricular function and/or hemodynamic stability persists suboptimal despite all therapies, mechanical circulatory support with intra-aortic balloon, extracorporeal membrane oxygenation, or ventricular assist device is indicated [56]. After MCS has been installed, new constant reevaluations should be done with attention to the new targets varying accordingly to the MCS chosen.

### Protocol validation

All selected judges were from Brazil, with participation of two women and four men. The judges appraised the protocol and successfully validated in all six domains with scores > 70% (scope and purpose, 94%; stakeholder involvement, 73%; rigor of development, 92%; clarity of presentation, 93%; applicability, 93%; editorial independence, 89%) using the selected AGREE II tool. Protocol overall quality achieved 89% and all judges recommended its use, with three judges recommending modifications. After discussion with all judges, we included all recommendations in the protocol.

All domain scores but one achieved approximately 90%. Domain 2 (stakeholder involvement) differed from all others by scoring 73%. The main reason, highlighted by the judges’ commentaries, was that the study aims to be developed by and to be directed only to anesthesiologists, without including other potentially interested professionals such as cardiologists or cardiac surgeons.

## Conclusion

The protocol development went through three major phases: systematic review, development, and validation. As a facilitating factor, we highlight that HTx anesthesiologists usually are a small homogenous group, thereby favoring implementations and enhancements over time. As barriers, we emphasize eventual drug or device shortages in HTx services and eventual low divulgation or practice of this protocol.

As limitations, we can highlight that there are no empirical data to link specific quality scores with specific implementation outcomes (e.g., speed and spread of adoption) or specific clinical outcomes; this makes the selection of quality thresholds to differentiate between high, moderate, and low-quality guidelines a challenge. Other limitations include the fact the only judges from one country were assessed.

We propose to periodically monitor and/or enhance the protocol every three to 5 years, or at such a time that new evidence or breakthroughs emerge in the medical literature. In the future, we intend to expand this protocol by involving worldwide professionals and a research group, which will include other stakeholder professionals (i.e., cardiologists, cardiac surgeons, intensivists).

We conclude that the protocol is validated and new studies are needed to assess its applicability and potential benefits on major endpoints.

## Data Availability

The datasets supporting the conclusions of this article are included within the article and its additional files.

## Abbreviations

BIS: Bispectral index
BiVAD: Biventricular assist device
CI: Cadiac index
CO: Cardiac output
CPB: Cardiopulmonary bypass
CVP: Central venous pressure
dIVC: Inferior vena cava distensibility index
DO_2_: Delivery of oxygen
ECMO: Extracorporeal membrane oxygenation
EKG: Electrocardiogram
EtCO_2_: End-tidal carbon dioxide
FAC: Fractional area chance
FiO_2_: Fraction of inspired oxygen
HR: Heart rate
HTx: Heart transplantations
IAB: Intra-aortic ballon
IBP: Invasive arterial blood pressure
iPC: Inhaled prostacyclin
iPDE-3: Phosphodiesterase type 3 inhibitors
ISHLT: International society for heart and lung transplantation
IVC: Inferior vena cava
IVC-CI: Inferior vena cava collapsibility index
LV: Left ventricle
LVAD: Left ventricular assist device
LVEF: Left ventricular ejection fraction
MAP: Mean arterial pressure
MCS: Mechanical circulatory support
MRI: Magnetic resonance imaging
NE: Norepinephrine
NO: Nitrous oxide
NPS: Nitroprusside
NTG: Nitroglycerin
PAC: Pulmonary arterial catheter
P_a_CO_2_: Arterial partial pressure of carbon dioxide
P_a_O_2_: Arterial partial pressure of oxygen
PAOP: Pulmonary artery occlusion pressure
PAP: Pulmonary arterial pressure
PCWP: Pulmonary capillary wedge pressure
PEEP: Positive end-expiratory pressure
PH: Pulmonary hypertension
P_plat_: Plateau pressure
PPV: Positive pressure ventilation
PVR: Pulmonary vascular resistance
RL: Ringer’s lactate
rSO_2_: Regional cerebral oxygen saturation
RV: Right ventricle
RVAD: Right ventricular assist device
RVD: Right ventricular dysfunction
RVEF: Right ventricle ejection fraction
S_a_O_2_: Arterial oxygen partial pressure
S_cv_O_2_: Mixed venous oxygen saturation
SpO_2_: Peripheral oxygen saturation
SV: Stroke volume
SVR: Systemic vascular resistence
TAPSE: Tricuspid annular plane systolic excursion
TEE: Transesophageal echocardiogram
UECE: Ceará State University
VP: Vasopressin
TV: Tidal volume
